# Harpin Hpa1 promotes flower development in *Impatiens* and *Parochetus* plants

**DOI:** 10.1186/s40529-016-0132-z

**Published:** 2016-08-11

**Authors:** Yilan Dong, Ping Li, Chunling Zhang

**Affiliations:** 1 College of Plant Protection, Nanjing Agricultural University, 1 Weigang Town, Nanjing, 210095 China; 2 Nanjing Foreign Language School, 30 East Beijing Road, Nanjing, 210008 China

**Keywords:** Hpa1_10-42_, *Impatiens* spp., *Parochetus communis*, Flowering, Anthocyanin

## Abstract

**Background:**

The harpin protein Hpa1 has multiple beneficial effects in plants, promoting plant growth and development, increasing crop yield, and inducing plant resistance to pathogens and insect pests. In these effects, the 10–40 residue fragment (Hpa1_10–42_) isolated from the Hpa1 sequence is 1.3 to 7.5-fold more effective than the full length.

**Results:**

This study extends the beneficial effects of Hpa1 and Hpa1_10–42_ to flower development in three species of the garden balsam *Impatiens* and the garden scoparius *Parochetus communis* plant. The external application of Hpa1 or Hpa1_10–42_ to the four ornamental plants had three effects, i.e., promoting flower growth, retarding senescence of fully expanded flowers, and increasing anthocyanin concentrations in those flowers and therefore improving their ornamental visages. Based on quantitative comparisons, Hpa1_10–42_ was at least 17 and 42 % more effective than Hpa1 to increase anthocyanin concentrations and to promote the growth of flowers or delay their senescence.

**Conclusion:**

Our results suggest that Hpa1 and especially Hpa1_10–42_ have a great potential of horticultural application to increase ornamental merits of the different garden plants.

## Background

Hpa1 is a harpin protein produced by *Xanthomonas oryzae*, the pathogen that causes bacterial blight of rice (Zhu et al. 2000; Liu et al. 2006; Chen et al. 2008a; Li et al. 2004, 2014a, b). Like all harpin orthologs identified in different species of Gram-negative plant pathogenic bacteria (Wei et al. 1992; He et al. 1993; Dong et al. 1999, 2004, 2005; Kim and Beer 2000; Liu et al. 2006, 2010, 2011), Hpa1 induces plant growth and defense responses (Peng et al. 2004; Liu et al. 2006; Ren et al. 2006a, b, 2008; Wu et al. 2007; Zhang et al. 2007, 2011a, b; Chen et al. 2008a, b; Sang et al. 2012). The dual effect depends on plant sensing of the nitroxyl-terminal region in the Hpa1 sequence (Wang et al. 2008; Li et al. 2014b; Ji et al. 2015). From this region, the 10–42 residue fragment (Hpa1_10–42_) has been isolated, produced by prokaryotic expression (Wu et al. 2007; Chen et al. 2008a; Li et al. 2014b), and analyzed for its multifaceted effects on Arabidopsis (biological model plant), tobacco (cash crop), tea (drinking crop), rice (food crop), and wheat (food crop). In these plants, Hpa1_10–42_ is 1.3 to 7.5-fold more effective than the full-length Hpa1 protein in inducing resistance to pathogens and enhancing plant growth or increasing crop products (Wu et al. 2007; Chen et al. 2008a, b; Li et al. 2014b). In addition to the phenotypic effects, Hpa1_10–42_ is also stronger than Hpa1 in inducing the expression of growth-promoting genes (Wu et al. 2007) and defense response genes (Chen et al. 2008a; Li et al. 2014b) in plants under conditions of normal growth and pathogen attack, respectively. Moreover, Hpa1_10–42_ display higher activities than Hpa1 in promoting leaf photosynthesis (Li et al. 2014b) and phytohormone signaling pathways (Chen et al. 2008a) associated correspondingly with plant growth enhancement and defense responses. These findings suggest the physiological and molecular bases of the multiple roles that Hpa1 and Hpa1_10–42_ play in plants.

These findings further suggest that Hpa1 and Hpa1_10–42_ in particular have a promising potential of agricultural application for improving developmental and/or defensive properties of economically significant crops. In tea plants, Hpa1_10–42_ is 1.3-fold more active than Hpa1 in increasing the yield of top three leaves used as drinking material (Wu et al. 2007). In rice, Hpa1_10–42_ is 2.7 and 7.5 times stronger than Hpa1 in eliciting resistance to blast (Chen et al. 2008b) and bacterial blight (Chen et al. 2008a), respectively. Meanwhile, the growth enhancement is 1.5-fold higher (Chen et al. 2008a) and the grain yield increase is 2.0-fold more (Chen et al. 2008b) in rice plants treated with Hpa1_10–42_ compared to Hpa1. In wheat, de novo expression of Hpa1_10–42_ leads to enhancements of resistance to *Fusarium* head blight (Yang et al. 2013), powdery mildew (Wang et al. 2014) and English grain aphid (Fu et al. 2014). In tobacco, however, Hpa1_10–42_ is near 30-fold less active than Hpa1 in eliciting the hypersensitive response (HR), which indicates the bioactivity of pathogen-derived compounds (Chen et al. 2008a; Wang et al. 2009). The HR associates with the induction of resistance to pathogens and is also a developmental cost associated with defense responses (Dangl et al. 1996; Yu et al. 1998; Peng et al. 2004). Indeed, resistance is activated in an HR-independent manner in Hpa1-expressing transgenic tobacco (Peng et al. 2004). Therefore, Hpa1_10–42_ is a desired agricultural agent that induces plant growth enhancement and defense responses with little cost of plant development (Peng et al. 2004; Wu et al. 2007; Chen et al. 2008a, b).

Based on the broad spectrum of the beneficial effects caused by Hpa1 and Hpa1_10–42_ in the biological model plant, as well as in the economically important cash, drinking, and food crops, we assumed that similar beneficial effects might be induced and employed to effectively increase ornamental merits of flowering horticultural plants. To test this idea, we chose to compare the effects of Hpa1 and Hpa1_10–42_ on flower development in garden balsam *Impatiens* spp. and garden scoparius *Parochetus communis* Buch.-Ham. ex D. Don.

## Methods

### Protein preparation

A previously described protocol (Chen et al. 2008a) was employed to prepare proteins used in this study. Proteins were produced by *Escherichia coli* cells transformed with the prokaryotic expression vector pET30a(+), namely empty vector, or the recombinant vector containing an insert of the *X. oryzae* *hpa1* gene or its truncated version *hpa1*_30–126_ coding for the Hpa1_10–42_ protein. Both *hpa1* and *hpa1*_30–126_ had been fused to the His tag encoding a peptide containing 6 histidine residues. The empty vector preparation (EVP) and the Hpa1-His or Hpa1_10–42_-His fusion protein preparation were purified by nickel chromatography and elution with aqueous imidazole solutions. Highly purified Hpa1-His protein was collected from the 200-mM imidazole eluent and used in the experiments after the His tag was removed by treatment with the Novagen Enterokinase Cleavage Capture Kit (EMD Biosciences Inc., Darmstadt, Germany). The 200-mM imidazole eluent of the EVP preparation was used as a negative control in the experiments. Proteins were fractioned by electrophoresis in tricine-sodium dodecyl sulphate-plolyacrylamide gel (T-SDS-PAGE) and visualized by gel staining with Coomassie Brilliant Blue G-250 (Schagger and Von Jagow 1987). Protein concentrations were determined (Dong et al. 1999); Hpa1 and Hpa1_10–42_ preparations were diluted with pure water; the EVP preparation was supplemented with bovine serum albumin (Bauer et al. 1995); and the final concentration of all protein preparations used in plant treatment was adjusted to 10 μg/ml, an active dosage under most circumstances (Peng et al. 2003; Liu et al. 2006; Chen et al. 2008a, b; Li et al. 2014a, b;). Before plant treatment, the surfactant Silwet-77 was added to the adjusted protein solutions at a concentration of 0.03 % to ensure uniform distribution of applied proteins on plant surfaces (Li et al. 2014a, b). The bioactivity of proteins was confirmed by testing the induction of HR in leaves of tobacco *Nicotiana benthamiana* L (Peng et al. 2003).

### Plant treatment and flower observation

Fifty-day-old *P. communis*, *I. balsamina*, *I. platypatala*, and *I. walleriana* plants from local market were transferred into plant growth chambers and grown under 24 ± 1 °C, 50 ± 2 % humidity, and short day (8-h light at 200 μM quanta/m^2^/s) conditions. Ten days later, aqueous protein solutions were applied separately by spraying over plant tops with the aid of atomizer. In the subsequent 1 month, morphological flowering characters were monitored, mainly including flower development stages, duration of each stage, and times to flower senescence. These parameters were determined on totally 300 uniform flowers selected from 30 plants assigned to three independent experiments (10 plants per experiment) for every combination between plant species and treatment. Flowers observed were labeled individually with plastic tabs hanged on flower stalks immediately before plant treatment. Diameters of the five most expanded flowers in a single plant were determined immediately before treatment and every 24 h in 6 days after treatment. Mean values of flower diameters were calculated based on determinations of 50 flowers in 10 plants to reflect extents of flower growth following different treatments.

### Anthocyanin measurement

The anthocyanin content in flowers was determined as previously described (Zhu et al. 2013). Total anthocyanin was extracted by homogenizing fresh flowers in liquid nitrogen. Fine flower powders were immediately lyophilized and maintained at −80 °C until use. Total anthocyanin in lyophilized flower powders was extracted by incubation in methanol solution containing 1 % hydrochloric acid for 18 h at room temperature and under moderate shaking. The extract suspension was centrifuged (12,000*g*, 4 °C, 1 min) to precipitate cellular debris and collect supernatant. Anthocyanin concentration in the supernatant was quantified by spectrophotometry and the endogenous content was scored in contrast to fresh weight of flowers used in the extract preparation.

### Data analysis

All experiments were carried out by completely randomized design and repeated at least three times with similar results. Quantitative data were analyzed with commercial IBM SPSS19.0 software package (IBM Corporation, Armonk, NY, USA; http://www-01.ibm.com/software/analytics/spss/). Homogeneity-of-variance in data was determined by Levene test, and formal distribution pattern of the data was confirmed by Kolmogorov–Smirnov test and P–P Plots, an SPSS tool that yields a graph to assess whether the data are normal or not (Shi 2012). Then, data were subjected to analysis of variance along with Fisher’s least significant difference test and Tukey–Kramer’s test (Ludbrook 1998), respectively, using the commercial SPSS19.0 software package.

## Results

### Hpa1 and Hpa1_10–42_ possess bioactivity

**Fig. 1:**
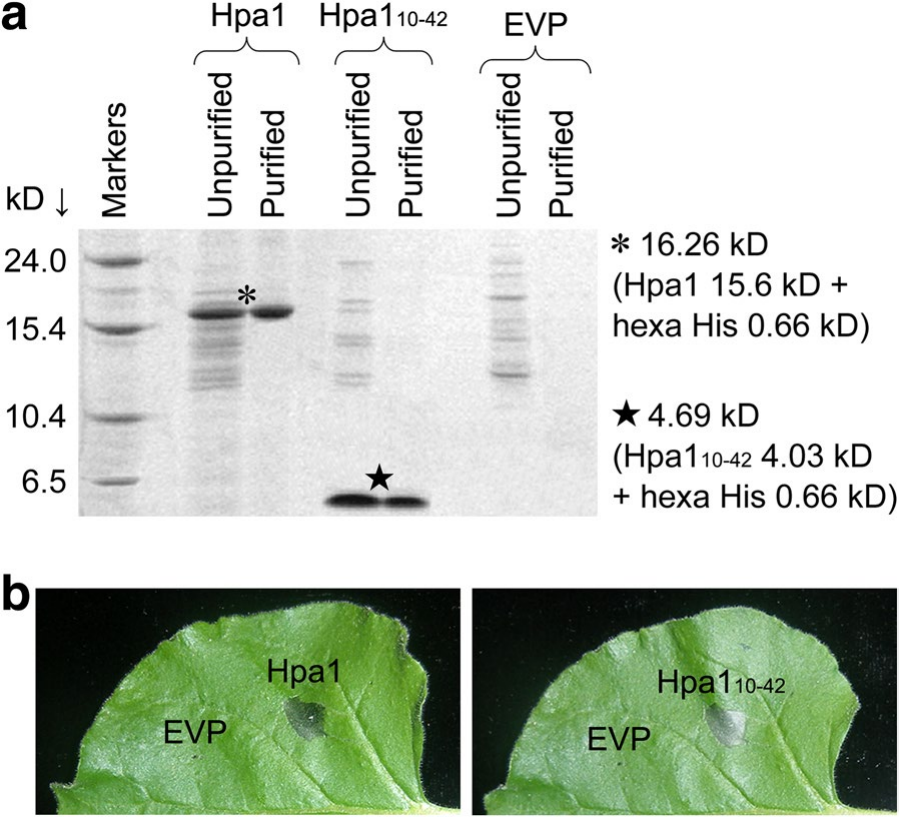
Purification and bioactive analyses of Hpa1 and Hpa1_10–42_ proteins in comparison with the control protein EVP. **a** Characterization of proteins by electrophoresis. The Hpa1-His and Hpa1_10–42_-His fusion proteins were produced after genetic combination in a prokaryotic expression vector. The empty vector preparation (EVP) was produced by using the vector without genetic combination. Proteins before and after purification were analyzed by electrophoresis in dodecyl sulphate-plolyacrylamide gel. Protein bands were visualized by gel staining with Coomassie Brilliant Blue G-250. **b** The bioactivity assay of proteins based on the induction of hypersensitive response (HR) in tobacco leaves. The his tag had been removed before the assay; aqueous solution of proteins were infiltrated into intercellular spaces of the leaves; leaves were photographed on 2 days after infiltration; and the HR appeared as local necrosis

After production by recombinant *E. coli* cells, the Hpa1-His and Hpa1_10–42_-His fusion proteins were purified and subjected to T-SDS-PAGE, which indicated that both fusion proteins were produced uniformly with correct sizes (Fig. 1a). The EVP sample contained inactive proteins but neither Hpa1-His nor Hpa1_10–42_-His; no proteins were detected from the EVP sample, which was subjected to a purification protocol similarly to Hpa1-His or Hpa1_10–42_-His (Fig. 1a). Based on the HR induction assay, a protocol that is widely employed to assess bioactivities of microbial proteins before application to plants (Peng et al. 2003; Li et al. 2004; Wang et al. 2009), EVP was inactive but both Hpa1 and Hpa1_10–42_ were active after His tag was removed (Fig. 1b). Thus, EVP was used properly as a negative control in the subsequent experiments. Because the HR-inducing activity of a microbial protein is associated with multiple effects including plant growth enhancement by the protein, purified Hpa1 and Hpa1_10–42_ are likely to have a growth-promoting effect in flowering horticultural plants.

### Hpa1_10–42_ and Hpa1 promote flower growth and extend flower maintenance period

**Fig. 2:**
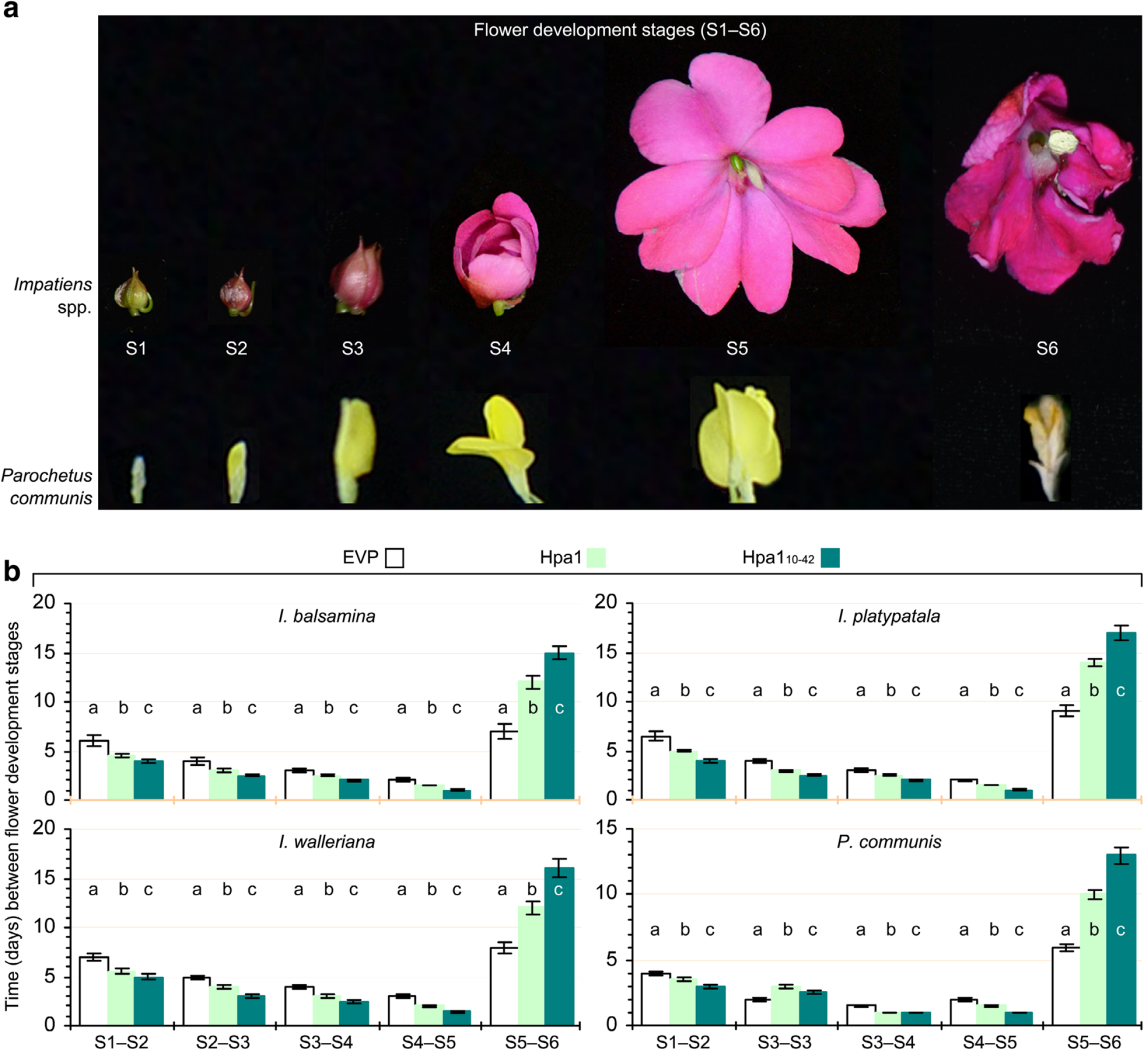
Flowers in development stages and the effects of Hpa1 and Hpa1_10–42_ on the progress of flower development. **a** Appearance of flowers at the different development stages (S1–S6). Showed flowers were from EVP-treated plants of the indicated species. **b** Times required for flower development in the indicated plants following treatment with EVP, Hpa1, or Hpa1_10–42_. Immediately before plant treatment, S1 flowers were labeled with roped plastic tabs for the subsequently tracked observations. Data shown are mean values ± standard deviation* bars* from three independent experiments each containing 300 flowers.* Different letters* in* bar graphs* indicate significant differences between treatments within the corresponding periods based on analysis of variance along with Fisher’s least significant difference test and Tukey–Kramer’s test (*n* = 300 flowers; *P* < 0.01)

In the three *Impatiens* species and in *P. communis* as well, flower development was divided into six stages, S1–S6 (Fig. 2a), to compare Hpa1 and Hpa1_10-42_ with EVP in terms of the effects on the morphological flowering character at every stage (Fig. 2b). Because flower buds were produced continuously at different times in a same plant individual and at a given time point the same plant had decades of flowers that fell into different development stages, uniform flowers were monitored since S1, the experimentally initial time point when flower buds had fully grown up but still appeared green (Fig. 2a). Subsequently, petals of a flower from S1 to S5 were growing circumstantially to allow for full opening of the flower at S5, followed by senescence that was easily to recognize (Fig. 2a). According to this chronological model, the normal process of flower development was monitored on plants assigned to the experimental control (EVP-treatment) group (Fig. 2a). All *Impatiens* plants performed similarly in the requirement for time to develop from S1 to S6, approximately taking 6, 4, 3, 2, and 9 days on average from S1 to S2, S2 to S3, S3 to S4, S4 to S5, and S5 to S6 in the order, whereas, times required for the corresponding five stage transitions were 4, 2, 1.5, 2, and 6 days in *P. communis* (Fig. 2b, empty black-line histograms). Therefore, efflorescent flowers well appeared in a maintenance period of 9 days in *Impatiens* spp. and 6 days in *P. communis* on average from full expansion at S5 to senescence at S6 under conditions of this study. Otherwise, the florescent maintenance period up to the end of S5 should merit the ornamental importance, which, however, must be lost circumstantially since flower senescence at S6.

The understanding of ornamental importance provides an angle to assess the effects of Hpa1 and Hpa1_10–42_ on flower development. In all plants, times needed for each of transitions from S1 to S5 were shortened but times required for the transition from S5 to S6 were extended significantly (*P* < 0.01) by the application of Hpa1 or Hpa1_10–42_ compared to EVP (Fig. 2b). This suggests that both Hpa1 and Hpa1_10–42_ had the activity to promote flower or petal growth and to extend the period of florescent maintenance. In total, the duration of S1–S5 was shortened approximately by 4 and 7 days in *Impatiens* spp. and by 1 and 2 days in *P. communis* plants following treatments with Hpa1 and Hpa1_10–42_, respectively. By contrast, Hpa1 and Hpa1_10–42_ treatments resulted in florescent maintenance period to be increased accordingly by 5 and 8 days in *Impatiens* spp. and by 4 and 8 days in *P. communis*.

**Fig. 3:**
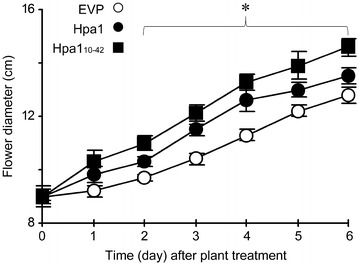
*P. communis* flower openings following the different treatments. Totally 10 plants were investigated for every treatment and diameters of the five most expanded flowers per plant were determined. Measurements of flower diameters were performed immediately before plant top spraying with aqueous solutions of the indicated compounds and at the indicated times. *Curves* represent mean values ± standard deviation; the *asterisk* indicates significant differences of data comparisons in the three treatments and in the range of the parenthesis (*P* < 0.05; *n* = 50 flowers)

Previously we have shown that plant growth enhancement becomes evident since 15 days after treatment with Hpa1 compared to EVP (Wu et al. 2007; Chen et al. 2008a, b; Li et al. 2014a, b). In previous studies, the growth-promoting effect of harpins took times to be detectable since the effect was evaluated by analyzing fresh weigh of vegetative organs or all aerial parts of plants. However, in the present study we found that the growth-promoting effect of Hpa1 and Hpa1_10–42_ was much earlier in flowers of *Impatiens* spp. *P. communis*. We found that Hpa1 and Hpa1_10–42_ treatments resulted in greater openings of flowers and this effect was evident since the second day of plant treatment. In the subsequent 4 days, flower diameters were up to 0.7and 1.1-cm greater in plants treated with Hpa1 and Hpa1_10–42_, respectively, than in EVP-treated plants (Fig. 3). This result and data shown in Fig. 2 suggest that Hpa1_10–42_ is more active than Hpa1 in the effects of promoting flower growth and extending florescent maintenance period.

### Hpa1_10–42_ and Hpa1 increase anthocyanin content in flowers

**Fig. 4:**
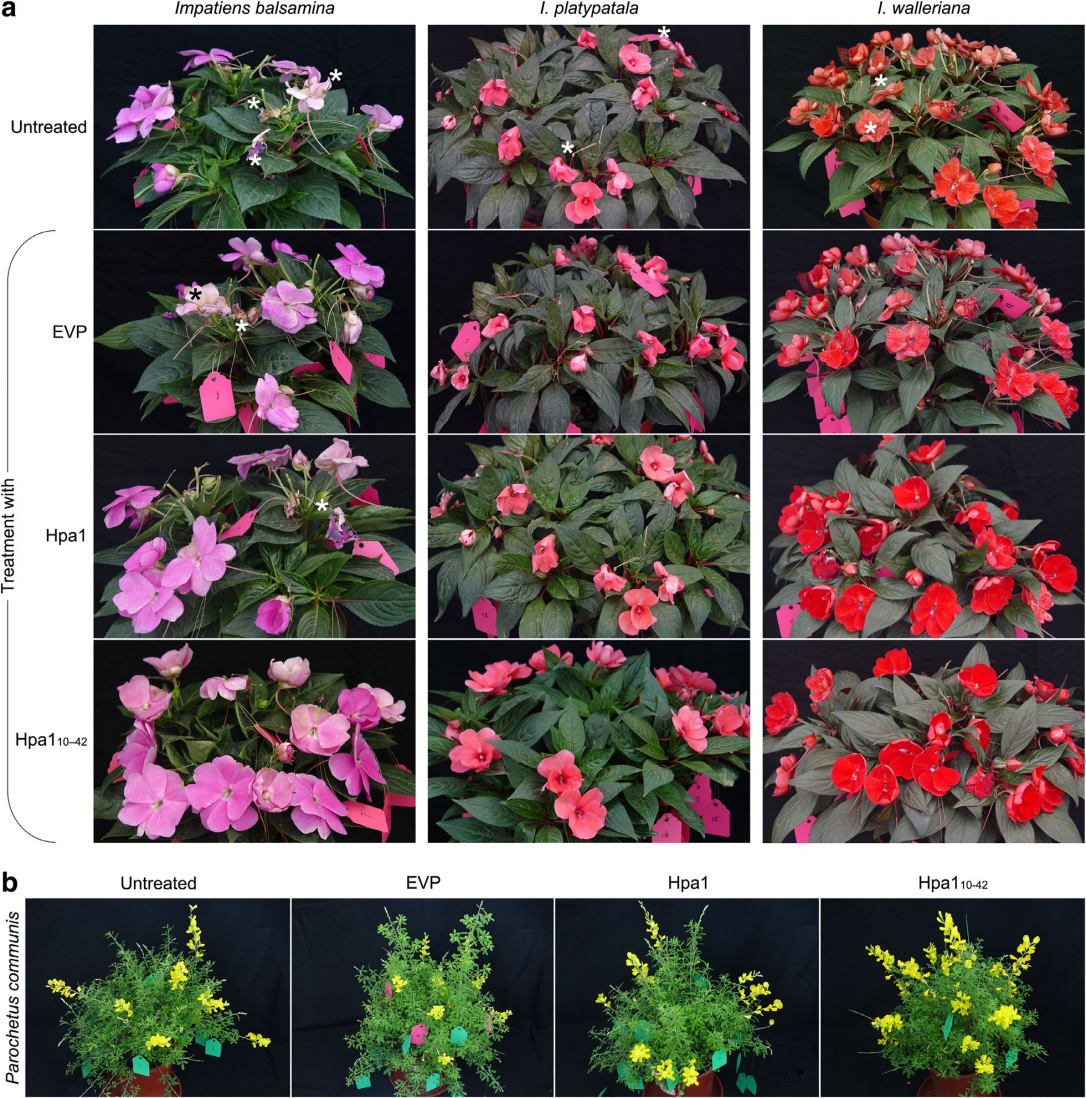
Flowering visages on 15 days after plant treatment as indicated. **a** *Impatiens* spp. Flowers that were experiencing senescence are indicated by *asterisks*. **b** *P. communis*

The overall effects of Hpa1_10–42_ and Hpa1 on the flowering phenotype were definitely observed on *Impatiens* spp. (Fig. 4a) and *P. communis* (Fig. 4b) plants photographed at 15 days after treatment. At that time, approximately 15 % flowers showed to fade in plants that were untreated or treated with EVP but not with Hpa1_10–42_ or Hpa1 except for a lower rate of flower senescence in Hpa1-treated *I. balsamina* plants (Fig. 4a, b), confirming the roles of Hpa1_10–42_ and Hpa1 in delaying flower senescence. In contrast to EVP, Hpa1_10–42_ or Hpa1 apparently improved flower qualities based on their color brilliance (Fig. 4a, b).

**Fig. 5:**
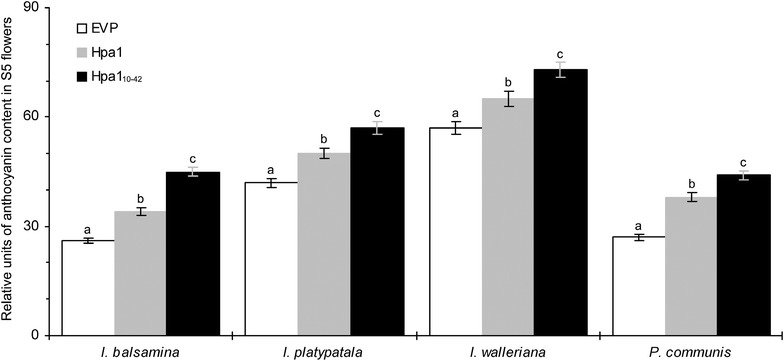
Relative levels of anthocyanin in S5 flowers of the differently treated plants. Anthocyanin was measured in flowers at the first day of S5. Data shown are mean values ± standard deviation bars from three independent experiments each containing five plants and 15 flowers.* Different letters* in *bar graphs* indicate significant differences between treatments within the corresponding periods based on analysis of variance along with Fisher’s least significant difference test and Tukey–Kramer’s test (*n* = 3 experiments; *P* < 0.01)

To infer the physiological basis for changes in the color brilliance of S5 flowers, we determined anthocyanin concentrations in S5 flowers of plants treated separately with EVP, Hpa1, and Hpa1_10–42_. We found that S5 flowers had significantly (*P* < 0.01) higher concentrations of anthocyanin in all plants treated with Hpa1 or Hpa1_10–42_ in contrast to EVP (Fig. 5). Under EVP treatment, relative levels of the steady-state anthocyanin were estimated to be 26, 42, 57, and 27 in S5 flowers of *I. balsamina*, *I. platypatala*, *I. walleriana*, and *P. communis*, respectively. Anthocyanin concentrations were markedly increased by Hpa1 or Hpa1_10–42_. In comparison, Hpa1 caused 14–31 % and 41 % increases of anthocyanin levels accordingly in S5 flowers of *Impatiens* spp. and *P. communis*, whereas, percentages of anthocyanin increases caused by Hpa1_10–42_ were 30–73 % in *Impatiens* spp. and 63 % in *P. communis*, respectively (Fig. 5). Therefore, Hpa1_10–42_ is more effective than Hpa1 in the effect of increasing anthocyanin content in S5 flowers.

## Discussion

The main purpose of this study was to determine whether the external application of Hpa1 and Hpa1_10–42_ to *P. communis*, *I. balsamina*, *I. platypatala*, and *I. walleriana* affects their morphologic flowering properties. We assumed that Hpa1 and Hpa1_10–42_ might function in horticultural plants as in biological model plants and crops. In the horticulturally ornamental plants, both proteins might promote flower growth, alter florescent duration or the period of flower maintenance (the period from flower initiation to senescence), and therefore improve ornamental merits of these horticultural plants. This hypothesis has been validated by data obtained from the replicate experiments. After application to balsam and scoparius plants, both Hpa1 and Hpa1_10–42_ effectively promote flower growth, retard senescence of fully expanded flowers, and increase anthocyanin quantities and therefore improve ornamental visages of flowers.

Both balsam and scoparius plants are very attractive models for analyses of flowering properties. They belong to short day plant, have the ability to continuously generate lateral shoots and to form floral organ primordia at the shoot apex, and can flower in all seasons under environment-controlled conditions (Cui 1998; Pouteau et al. 1998; Tooke et al. 2005). In the garden balsam plants, moreover, floral organs and petals in particular are characteristic of mosaic colors, from white to salmon pink, pink, red, or purple (Pouteau et al. 1998; Tooke et al. 2005) due to different concentrations of the pigment anthocyanin and changes of cellular pH conditions (Hagen 1959; Asen et al. 1972). This well-studied morphological and physiological relationship (Zhu et al. 2013) allows for convenient assessments of flowering qualities in terms of ornamental merits. While *Parochetus communis* is the only scoparius species currently available in the local market, three *Impatiens* species, *I. balsamina* L., *I. platypatala* L., and *I. walleriana* Hook. f. (Jarvis 2007; Christenhusz 2010), are popularly planted as horticultural crops in China (Yu 2012).

The effects of Hpa1 and Hpa1_10-42_ on the garden plants result in apparently improved qualities of flowers and presumably also increase the ornamental value of these flowering horticulture plants. Quite consistent with previous studies that have demonstrated the vigorous effect of Hpa1_10–42_ (Wu et al. 2007; Chen et al. 2008a, b; Li et al. 2014b), this truncated Hpa1 fragment is more effective than the full-length protein in the triple effects and possibly in the potential ornamental importance as well. Furthermore, it has been found that harpins travel across plant cell walls and finally localize to the plasma membrane, followed by cellular responses (Oh and Beer 2007; Sang et al. 2012; Li et al. 2015; Tian et al. 2016). Because plant cell walls are highly porous and cannot block passage of large molecules, including proteins, no matter how a harpin gets access to plant surfaces, it should smoothly traverse cell walls, associate with plasma membranes, and activate cellular responses. This provides the molecular basis for the agricultural application of Hpa1 and Hpa1_10–42_ in crop improvements and also in increasing the ornamental value of horticulture plants. However, we did not analyze any molecular mechanisms by which Hpa1 and Hpa1_10–42_ affect the flower development, and this will be the subject for further studies.

In addition to plant sensing of harpins, the subsequently activated physiological and pathological responses are critical for the developmental and immune roles that harpins play in a variety of plants (Li et al. 2015; Tian et al. 2016). And this functional relationship is determined by the biochemical characteristics of harpins as type III accessory proteins (Ji and Dong 2015). Harpins belong to a unique group of proteins secreted by the type III secretion system in plant-pathogenic Gram-negative bacteria (Zhu et al. 2000; Choi et al. 2013; Ji and Dong 2015). To date, totally 23 harpins have been identified in different bacterial species and are divided into one-domain and two-domain harpins based on the unitary hydrophilic domain and an additional enzymatic domain (Choi et al. 2013; Ji and Dong 2015). While two-domain harpins potentially associate with the bacterial periplasm or plant cell walls to facilitate assembly of the secretion machinery, one-domain harpins target plasma membranes to cause three distinct biological effects in a variety of plant species (Oh and Beer 2007; Chen et al. 2008a, b; Sang et al. 2012; Wang et al. 2014; Li et al. 2015). Hpa1 is a one-domain harpin and performs a full repertoire of functions shared by all harpins tested so far (Zhu et al. 2000; Chen et al. 2008a, b; Li et al. 2014a, b, 2015; Ji and Dong 2015). One-domain harpins are the jack of all bacterial proteins secreted by the type III secretion system, with the critical effects on bacterial virulence to host plants and both growth and immunity enhancements of nonhosts in a pathogen-independent manner (Choi et al. 2013; Ji and Dong 2015; Tian et al. 2016). The multiple functions of one-domain harpins, including Hpa1, depend on the activation of distinct signaling pathways in biological model plants and crops (Chen et al. 2008a, b; Choi et al. 2013; Li et al. 2014a; 2015; Tian et al. 2016). In these plants, signaling pathways responsible for plant growth or immunity have been well demonstrated with sufficient information on the plant genomes. However, genomic information on *P. communis*, *I. balsamina*, *I. platypatala*, and *I. walleriana* is now totally absent. Thus, an expected role of the present study would be to stimulate studies in the future to elucidate genetic and molecular mechanisms that underpin the beneficial effects of Hpa1 and Hpa1_10–42_ in the horticulturally important ornamental plants.

## Conclusion

The application of Hpa1 and Hpa1_10–42_ promotes flower growth and expansion, retards senescence of fully expanded flowers, extends their florescent period, and increases concentrations of anthocyanin in fully expanded flowers in garden balsam *Impatiens* spp. and garden scoparius *Parochetus communis*. This finding offers an effective way to increase ornamental merits of the different garden plants.

## Abbreviations

HR: hypersensitive response
Hpa1: HR and pathogenicity-associated 1
EVP: the empty vector preparation
